# Mai kāpae i ke a’o a ka makua, aia he ola ma laila: Shifting Power through Hawaiian Language Reclamation

**DOI:** 10.3390/genealogy8030118

**Published:** 2024-09-13

**Authors:** Justin Kepo’o Keli’ipa’akaua, Shelley Muneoka, Kathryn L. Braun

**Affiliations:** Hā Kūpuna National Resource Center for Native Hawaiian Elders, Thompson School of Social Work & Public Health, University of Hawai’i at Mānoa, Honolulu, HI 96822, USA

**Keywords:** eldercare, language reclamation, kupuna, kūpuna, Native elders, ‘Ōlelo Hawai’i, Hawaiian literacy, power

## Abstract

Language loss hinders the expression of Indigenous Peoples and their unique worldviews, impairing the intergenerational transfer of knowledge. In Hawai’i, where a vast majority of the population was fluent and “universally literate” in ‘Ōlelo Hawai’i from the mid to late 1800s, colonial impositions drastically reduced the number of fluent speakers to roughly 2000 by the 1970s. Efforts to revitalize the language since then have greatly increased the number of current ‘Ōlelo Hawai’i speakers and resources. Building upon this great work, the Hā Kūpuna National Resource Center for Native Hawaiian Elders at the University of Hawai’i has initiated projects to contribute to the reclamation of ‘Ōlelo Hawai’i by increasing our contemporary understanding of ancestral Hawaiian perspectives on elders. To support these projects, significant changes in power structures within our organization were necessary. Insights gained from these projects include gaining clarity on the evolution of the usage of the word “kupuna”, identifying more nuanced perspectives on elders, understanding the importance of family relationships on caregiving outcomes, and understanding the importance of carefully translating English words into ‘Ōlelo Hawai’i.

## 1. Introduction

Language loss following colonization compromises the personal identity of Indigenous Peoples, hinders the expression of a culture’s unique worldview, and impairs the intergenerational transfer of knowledge, including knowledge related to living in harmony with the local environment. The cumulative effects of assimilation have contributed to poor mental and physical health outcomes among Indigenous people, as well as environmental degradation. In Hawai’i, the majority of the Indigenous population was literate in ‘Ōlelo Hawai’i (the Hawaiian language) starting in the 1820s, up until which the language was oral-aural only. Following the illegal overthrow of the Hawaiian Kingdom in 1893, and the imposition of the English language as the medium of instruction in public education in 1896, the number of fluent speakers of ‘Ōlelo Hawai’i dropped precipitously, and by the late 1970s, it was estimated that there were only 2000 native speakers of ‘Ōlelo Hawai’i remaining (Warner 2001, p. 135).

Since the 1960s, great efforts to revitalize the language have resulted in more speakers of ‘Ōlelo Hawai’i and more ‘Ōlelo Hawai’i resources available to these speakers. Language reclamation has been found to improve individual and community well-being. In a systematic review of 130 publications reporting on qualitative and quantitative explorations of language use and health outcomes, Whalen et al. (2022) found good evidence that active use and reclamation of an Indigenous language has positive benefits for well-being, health, and general fitness, and is a protective factor against poor health. Research into ‘Ōlelo Hawai’i source documents allows us to connect to the wisdom and perspectives of our Native Hawaiian ancestors. This process grants us insights into our own identity, allowing us to be transitional characters in our own growth and healing in ways that resist and counter generational patterns of trauma (Hemphill 2024, pp. xxiv–xxv) stemming from Native Hawaiian displacement from land, government, and language since 1893. This paper summarizes current efforts to help reclaim ‘Ōlelo Hawai’i by the Hā Kūpuna National Resource Center for Native Hawaiian Elders at the University of Hawai’i. Insights gained from several Hā Kūpuna projects, including two collections of words and sayings related to aging and an ‘Ōlelo Hawai’i storybook about dementia for youth, are discussed following a brief history of language loss and resurgence in Hawai’i. The fourth section of this paper describes shifts in power structures within Hā Kūpuna, a resource center housed within a State University, that enabled these projects to be supported. Hawaiian language words and terms are included in this paper without the use of italic or bold font, but are highlighted in specific instances with the use of quotation marks (“ “) to indicate key terms identified during our projects for illustrating the importance of carefully translating English terms into ‘Ōlelo Hawai’i.

## 2. Brief History of Language Loss and Resurgence

Since time immemorial, ‘Ōlelo Hawai’i was the primary language spoken in Hawai’i. From the 1830s to the 1860s, a large portion of the population of the Kingdom of Hawai’i was literate, with many people able to read and write not only in ‘Ōlelo Hawai’i, but in English, as well. By the 1900s however, fluency in ‘Ōlelo Hawai’i started to decline drastically. Beginning in the 1960s, a resurgence in Hawaiian culture and language spurred a renaissance resulting in the continued growth of fluent Hawaiian language speakers today. This section briefly details the history of language loss and resurgence which is important for contextualizing Hā Kūpuna’s current Hawaiian language projects.

### 2.1. The Hawaiian Kingdom, a Kingdom of Literacy—1820s to 1860s

“O ko’u aupuni, he aupuni palapala ko’u…No laila, e nā ali’i a me nā maka’āinana, e a’o ‘oukou i ka palapala”

My kingdom is a kingdom of literacy…Therefore, chiefs and commoners, you should all learn to read and write.

Kauikeaouli, Kamehameha III (Kamakau in Nogelmeier 2010, p. 68)

The written form of ‘Ōlelo Hawai’i was developed by Hawaiians and Calvinist missionaries in 1820 (Schütz in Nogelmeier 2010, p. 68). By 1824, most ruling chiefs were supporting schools in their lands and actively urged their people to attend classes and learn the pī’āpā, or alphabet for reading and writing (Kamakau in Nogelmeier 2010, p. 68). Kauikeaouli, Kamehameha III (the head of state of the Hawaiian Kingdom) involved himself personally in fostering education, with the ruling chiefs under him following his lead, including the model he set through his own attendance in class (Nogelmeier 2010, p. 68). Literacy was so commonplace that the 19th century scholar, Samuel Mānaiakalani Kamakau, remarked that by the mid-1820s one could ask, “Aia la mahea ka poe ike ole i ka heluhelu?” [Where would one find folks who do not know how to read?] (Kamakau in Nogelmeier 2010, p. 71).

By 1834, missionaries reported that 20,000 Native Hawaiians, roughly one-fifth of the population, actively attended schools at a ratio of three adults to each child (Schütz in Nogelmeier 2010, p. 71). In 1848, 19,644 students were enrolled in 624 schools conducted in the Hawaiian language, comprising 99% of total school enrollment (200 students enrolled in seven English schools comprised the remaining 1%) (Reinecke 1969, p. 70) (Table 1). Accounts of the extent of literacy vary, from 91% of Hawai’i’s population in 1832 (Laimana Jr. 2011, p. 166), three-fourths of all Hawai’i residents aged 16 and older in 1853 (Schmitt 1977, p. 209), and “nearly universal” by 1861 (Silva 2004, p. 55). Hawai’i boasted nearly 100 different newspapers published in ‘Ōlelo Hawai’i from 1834 to 1948 (referred to commonly as the nūpepa Hawai’i, or Hawaiian language newspapers), comprising roughly 125,000 pages, which if printed on letter-sized paper would exceed one million pages of typescript text (Nogelmeier 2003, 2010, p. 64).

### 2.2. ‘Ōlelo Hawai’i in Decline—1860s to 1902s

Twenty years later, in 1868, enrollment in Hawaiian language schools had declined to 6323 students in 221 Hawaiian schools, comprising 75% of total school enrollment. By 1888 these numbers declined drastically to 1370 students in 63 Hawaiian schools, comprising 15.7% of total school enrollment (Reinecke 1969, p. 71). The illegal overthrow of the Hawaiian Kingdom government in 1893 (U. S. Congress 1993; U. S. House of Representatives and Foreign Relations Committee 1894, pp. 445–58) led by treasonous plantation owners and backed by the U. S. military displaced the Native Hawaiian Queen and usurped rule of the kingdom. By 1896, the same illegal government effectively displaced the Hawaiian language, along with all other languages previously spoken in the Kingdom of Hawai’i, when English was imposed as the only allowable medium of instruction in schools (Kimura 1989, p. 74; Warner 2001, p. 134). By 1897, 26 students were enrolled in one Hawaiian school comprising 0.2% of total school enrollment. By 1902, zero students were enrolled in zero Hawaiian schools comprising 0% of total school enrollment (Reinecke 1969, p. 72) (Table 1).

### 2.3. The Resurgence of Hawaiian Language and Culture—1960s until Present

In the late 1960s, a cultural revolution centered in Hawaiian music and dance occurred among young Hawaiians (Warner 2001, p. 135). This led to a resurgence in Hawaiian language and culture in the mid to late 1970s, known commonly as the Hawaiian Renaissance (Warner 2001; Muneoka et al. 2021). In the 1978 constitutional convention, the Hawaiian language was designated as one of two official languages of the state of Hawai’i along with English, and a separate law designated Hawaiian as the official native language of the state (Warner 2001, p. 135). A survey conducted that same year estimated that there were only 2000 native speakers of ‘Ōlelo Hawai’i remaining.

By 1982, the number of second-language speakers of ‘Ōlelo Hawai’i was estimated at 150 to 200, and it became apparent that for the language to survive, a new generation of speakers was needed. Inspired by the Kōhanga Reo (Māori language immersion preschools), the first Pūnana Leo (“language nest”) Hawaiian language immersion preschool opened in 1984 in Kekaha, Kaua’i. Two more were opened in Hilo, Hawai’i and Kalihi, O’ahu in 1985. By 1999, there were eleven Pūnana Leo serving 209 children (Kimura 1989, p. 75; Warner 2001, pp. 135–37). By 1992, the Kula Kaiapuni (Hawaiian Immersion Schools) and the Hawaiian Language Immersion Program were expanded to an intensive kindergarten to 12th grade model. By 1999, the total enrollment of the Kula Kaiapuni program was 1543 K-12 students (Wilhelm in Warner 2001, p. 140). Combined with students at the Pūnana Leo, 1760 students were being instructed through ‘Ōlelo Hawai’i in 1999 (Warner 2001, p. 140)—a number unheard of since the 1880s. At the collegiate level, bachelor’s and master’s degrees in ‘Ōlelo Hawai’i are currently offered at the Kawaihuelani Center for Hawaiian Language at the University of Hawai’i at Mānoa, while bachelor’s, master’s, and doctoral degrees are offered through the Ka Haka ‘Ula o Ke’elikōlani Hawaiian Language College at the University of Hawai’i at Hilo.

In 2001, Ho’olaupa’i began as a pilot project that has since digitized over 15,000 pages of nūpepa Hawai’i, making images of these nūpepa and over 150,000 searchable pages of typescript publicly accessible (Nogelmeier 2010, pp. 161–62). Digital copies of these nūpepa are currently available on online databases like Ulukau (Hale Kuamo’o 2024), and the Papakilo Database (Office of Hawaiian Affairs (OHA) (2024)). The recent establishment of the Ke’ena Noi’i a Unuhi ‘Ōlelo Hawai’i (Institute for Hawaiian Language Research and Translation) contributes to increasing access to information found in these nūpepa and many other Hawaiian language source materials. Awaiaulu, an organization formed in 2004, has been crucial in producing accessible resources from Hawaiian language source materials and in training new Hawaiian language translators and editors. These efforts have greatly increased accessibility to a large repository of Hawaiian language source material for families, scholars, and professionals alike, and has made possible the current Hawaiian language projects undertaken by the Hā Kūpuna National Resource Center.

## 3. Hā Kūpuna Projects Aimed at Language Reclamation

The Hā Kūpuna National Resource Center (NRC) for Native Hawaiian Elders is one of three NRCs for Native Elders funded by the U. S. Administration on Aging, Department of Health and Human Services. Hā Kūpuna is housed under the Thompson School of Social Work at the University of Hawai’i at Mānoa. Other NRCs are the National Resource Center on Native American Aging at the University of North Dakota and the National Resource Center for Alaska Natives at the University of Alaska Anchorage. The main task of these NRCs is to use research to support elders and the organizations and individuals who provide eldercare. A majority of Hā Kūpuna’s research has focused on data collection and analysis and oral history projects, publishing the findings through scholarly articles and through fact sheets that are more accessible to the community at large. The fact sheets are available for free on our website, unlike many journal articles that are typically available only via purchase; additionally, the fact sheets refrain from using scholarly jargon and prioritize language that is understandable to most people outside of academia, including the elders for whom this research is meant to serve.

As a part of our ongoing research to support Native Hawaiian elders, Hā Kūpuna has recently embarked on multiple projects that prioritize ‘Ōlelo Hawai’i. One goal of these projects is to contribute to reclaiming Hawaiian identity through understanding our own cultural perspectives on elders and eldercare. Additionally, we aim to increase the availability of culturally grounded and sensitive care for Native Hawaiian elders, by conducting and disseminating research on Hawaiian knowledge and practices pertaining to later life. These efforts also were in support of initiatives to make the University of Hawai’i a “Hawaiian Place of Learning” (Lipe 2016). Our Hawaiian language projects also support the Department of Social Work’s unique 10th competency, which calls on students to “Engage, honor, and respect Indigenous culture towards decolonized professional practice”, and includes a specific mention of respect for traditional ways of knowing (Nakaoka et al. 2019). This decolonial process contributes to language reclamation by centering our language projects on community needs and perspectives (Leonard 2017, pp. 19–20).

Hā Kūpuna’s Hawaiian language projects either rely primarily on ‘Ōlelo Hawai’i resources or translate English language resources into ‘Ōlelo Hawai’i. What began as a query into the digital repository of nūpepa Hawai’i for insights into ancestral Hawaiian perspectives and practices pertaining to elders and eldercare, expanded into the development of two ‘Ōlelo Hawai’i collections that serve as useful resources in their own right. One collection is a glossary of Hawaiian language terms pertaining to elderhood, and the other is a collection of Hawaiian proverbs, poetical sayings, and riddles pertaining to elderhood. The third language project is a translation of our dementia storybook, *Pōmai and Her Papa,* from English into ‘Ōlelo Hawai’i. These projects are described in more detail in this section along with insights gained from this research.

### 3.1. E Ola ā Kau ā Kaniko’o: A Glossary of Hawaiian Language Terms Pertaining to Elderhood

The glossary of Hawaiian terms pertaining to elders, titled E Ola ā Kau ā Kaniko’o: He ‘Ohina Hua ‘Ōlelo no nā Kūpuna, Luāhine me nā ‘Elemākule; A Glossary of Hawaiian Language Terms Pertaining to Elderhood (Keli’ipa’akaua et al. 2023), was compiled after beginning initial queries into the online databases of nūpepa Hawai’i for insights into ancestral Hawaiian perspectives on elders and eldercare. In querying these databases, we began to assemble a list of search terms. This list of search terms proved to be quite extensive, at which point it was decided to compile the terms into an organized glossary as the breadth of terms, each with multiple definitions, proved to be a valuable resource in itself for understanding Hawaiian perspectives on elders and elderhood.

The finished glossary contains 231 terms, gathered through research and with definitions from the 1986 Hawaiian Dictionary by Mary Kawena Pukui and Samuel Elbert (Pukui and Elbert 1986) and the 1865 Dictionary of the Hawaiian Language prepared by Reverand Lorrin Andrews (Andrews 1865). The glossary contains an index organizing the glossary entries into 33 themes and 27 sub-themes that include topics like stages of life, physical condition, hair color, gender specific and gender-neutral terms, and others. The glossary is available for free as a digital PDF on our website at https://manoa.hawaii.edu/hakupuna/glossary-and-%CA%BBolelo-no%CA%BBeau-collection/.

### 3.2. E Ola ā Kau Kō Kea: A Collection of Hawaiian Proverbs, Poetic Sayings, and Riddles Pertaining to Elderhood

The ‘Ōlelo No’eau collection, titled E Ola ā Kau Kō Kea: He ‘Ohina ‘Ōlelo No’eau a Nane no nā Kūpuna, nā Luāhine me nā ‘Elemākule; A Collection of Hawaiian Proverbs, Poetic Sayings, and Riddles Pertaining to Elderhood (Muneoka et al. 2023), was compiled to identify additional proverbs and sayings pertaining to elders beyond the few more commonly known epithets used in prayers and songs that ask for and celebrate long life. It is our hope that readers of this collection will become familiar with and begin to use once again the poetic references to elders and aging that were once commonly understood. This collection contains 109 sayings or riddles relevant to ancestors, elders, aging, and elderhood that have been pulled from Handy and Pukui’s The Polynesian Family System in Ka’ū (Handy and Pukui [1958] 1998), Judd’s Hawaiian Proverbs and Riddles (Judd [1930] 1978), and Pukui’s seminal work on the topic ‘Ōlelo No’eau: Hawaiian Proverbs and Poetical Sayings (Pukui [1983] 1997). Like the glossary, this collection is divided into themes (25 total) and sub-themes (10 total) that cover similar topics to the glossary’s index, with the inclusion of a few others like “jokingly used references”, “unflattering sayings”, and “caregiving”. The ‘Ōlelo No’eau collection is also available for free as a digital PDF on our website at https://manoa.hawaii.edu/hakupuna/glossary-and-%CA%BBolelo-no%CA%BBeau-collection/.

### 3.3. Pōmai Lāua ‘o Papa: A Storybook for Learning About Dementia

Pōmai Lāua ‘o Papa; Ka Wā Kamali’i a me ka Makua Poina Wale: Pehea e Mālama ai i nā Mea Ko’iko’i loa (Hā Kūpuna ke Kikowaena Noi’i Kāko’o ma hope o nā Kūpuna Kānaka ‘Ōiwi Hawai’i 2023) is the ‘Ōlelo Hawai’i translation of the original book, Pōmai and Her Papa: Growing Up Around Memory Loss and Holding on to What Matters Most (Hā Kūpuna National Resource Center for Native Hawaiian Elders 2021). Both books are aimed at helping families to recognize signs of Alzheimer’s Disease and dementia, differentiating these from normal signs of aging, and to identify professionals and organizations who can assist in diagnosing and caring for elders with dementia. The translation of this book into ‘Ōlelo Hawai’i was done at the urging of our Joint Advisory Committee to provide more Hawaiian language eldercare resources from our NRC in consideration of the rising number of ‘Ōlelo Hawai’i speakers. For this translation, new words and terms were created for English terms, titles, and jargon for which Hawaiian language translations did not already exist (e.g., social worker, geriatrician, dementia). Both English and ‘Ōlelo Hawai’i versions of this storybook are available for free as a digital PDF on our website at https://manoa.hawaii.edu/hakupuna/pomai-and-her-papa/.

### 3.4. Insights Gained from the ‘Ōlelo Hawai’i Projects

Hā Kūpuna’s current Hawaiian language projects have yielded important insights into ancestral Hawaiian perspectives on elders and eldercare that contribute to this niche aspect of reclaiming Hawaiian identity. These insights include gaining clarity on the usage of the word “kupuna”, which is the hua ‘Ōlelo Hawai’i (Hawaiian word) commonly used for elders today, identifying nuanced perspectives on elders that run counter to the predominantly held reverent notions of elders, understanding the importance of family as it relates to caregiving, and understanding the importance of carefully translating English words into ‘Ōlelo Hawai’i. These four primary insights gained are described in more detail in this section.

#### 3.4.1. Evolution in the Usage of the Word “Kupuna”

One key takeaway from this research was insight into the evolution of the usage of the word “kupuna”. Both the Pukui and Elbert dictionary and the Andrews dictionary define the word kupuna in reference to an elder, either a grandparent, or one of an older generation to whom one specifically has a familial or close social relationship with (Pukui and Elbert 1986, p. 186; Andrews 1865, p. 320). By contrast, today kupuna (or kūpuna when pluralized) is used colloquially to refer to any older person, like the English term “senior citizen”, and has lost the added distinction of an elder or ancestor with whom one has a familial relationship with. Words that were commonly used for elders in general include the word luahine, used for female elders (Pukui and Elbert 1986, p. 213; Andrews 1865, pp. 351, 356), ‘elemakule, used primarily for male elders, but also used in plural when referring to a mixed group of elders (Pukui and Elbert 1986, p. 40; Andrews 1865, p. 69, Keli’ipa’akaua et al. 2023, p. 4), and makule, used to refer to elderly people in general (Pukui and Elbert 1986, p. 231), among other terms.

#### 3.4.2. Derisive References to Elders

An insight gained that runs somewhat counter to predominant contemporary notions pertaining to elders is found in the use of derisive terms. Today, elders are generally held with a sense of respect, reverence, and honor as reflected in terms like hulu (esteemed, choice, precious; esteemed older relative as of parents or grandparents’ generation) (Pukui and Elbert 1986, p. 89) and hulu kupuna (the precious few living blood relatives of the grandparents’ generation) (Pukui and Elbert 1986, p. 90). However, there are terms and phrases that point out that being older does not, in and of itself, entitle one to respect, while other terms and phrases are used in jest or are plainly insulting.

The word hāwena, as seen from examples in both collections, describes how age alone does not grant one knowledge or perhaps even the respect that accompanies such wisdom. A few definitions for hāwena include “White lime as used for dressing hair and turning the hair brown”, and another use of the word is “applied to a gray headed man [Ed. person] who has but little wisdom;” (Pukui and Elbert 1986, p. 62; Andrews 1865, p. 153). An example of the use of the word hāwena is found in the following ‘Ōlelo No’eau:

**Hāpala ‘ia a’e la i ka hāwena.** *Daubed with lime*.

Said of an oldster without wisdom. His hair may be gray, as one whose hair is bleached with lime, but he has no more wisdom than an inexperienced youth.

(Pukui [1983] 1997, p. 58, #483)

Words used in outright insulting or jesting ways include terms like ku’i lena (molar tooth yellow with age; insulting reference to old person) (Pukui and Elbert 1986, p. 175), and nenanena ‘auwae (yellowed jaws; insult to old men [Ed. person]) (Pukui and Elbert 1986, p. 264; Elbert and Pukui 1979, p. 25). ‘Ōlelo No’eau that are insulting to elders also exist as in the following example:

**Hele a ‘īlio pī’alu ka uka o Hāmākua i ka lā.** Like a wrinkled dog in the upland of Hāmākua in the sunlight.

An uncomplimentary remark about an aged, wrinkled person. Line from a chant.

(Pukui [1983] 1997, p. 80, #728)

Here we see another example of how the two collections complement each other in helping us to get a better understanding of these words and phrases. The word pī’alu, which may be used to plainly describe wrinkling with age, as with sagging eyelids or jowls (Pukui and Elbert 1986, p. 326), also means “to be heavy, as the eyes; to be almost blind, as an aged person” (Andrews 1865, p. 461). This word is used in the above ‘Ōlelo No’eau with an intentionally insulting tone, and therefore informs us about its selective use as a derisive term.

#### 3.4.3. Family and Caregiving

The importance of family and the reciprocity of care are concepts that have been important within Hawaiian society from the beginning of time and are evident in genealogies and mo’olelo (stories, traditions, histories) (Pukui and Elbert 1986, p. 254; Kame’eleihiwa 1992). Through our projects we identified a few specific ways in which these reciprocal family relationships are represented in relation to elders, and some implications that these relationships may have for eldercare.

Aside from the word kupuna, other words that describe being a grandparent include hi’imo’opuna (to bear a grandchild in the arms; to be a grandparent; a term of pride and affection) (Pukui and Elbert 1986, p. 68) and kanimo’opuna which refers to the sound of a grandchild’s wail (Pukui and Elbert 1986, p. 259). Each of these endearing words referring to grandparents contextualizes their role by centering the younger family member, the mo’opuna (grandchild), to define the role of the elder. While these two terms utilize the word for grandchildren to describe and to honor grandparents, there are other expressions that invoke the grandparents honoring a grandchild. The reciprocal nature of this endearing relationship is shown in the following ‘Ōlelo No’eau:

**He keiki mea kupuna.** [It shows] that the child has a grandparent.

Said in admiration of a child whose grandparents show affection by making beautiful things for his use or compose songs and chants in his honor. A similar expression is He keiki mea makua: [It shows] that the child has a parent.

(Pukui [1983] 1997, p. 77, #688)

The importance of maintaining healthy parent–child and grandparent–grandchild relationships manifest in ‘Ōlelo No’eau that speak to caregiving relationships.

**Ua ‘ai au i kāna loa’a**. I have eaten of his gain.

Said with pride and affection by a parent or grandparent who is being cared for by the children he reared.

(Pukui [1983] 1997, p. 305, #2769)

The ‘Ōlelo No’eau above speaks to the pride and perhaps security one feels when they are cared for by children they raised. Additionally, an elder may feel a sense of accomplishment in seeing the skills and values they’ve instilled in their children reflected back to them through their success and aloha. This reciprocity is especially important when we consider words used for caregiving. The word hānai is defined as “to raise, rear, feed, nourish, sustain; provider, caretaker” (Pukui and Elbert 1986, p. 56) and as “one fed or sustained by another” (Andrews 1865, p. 148). This word is frequently used to describe the care one gives to children, though it seems it may also be fitting in providing care for elders. Hānai, in its most basic definition, means to feed. When we consider another word, pū’ā (to feed by passing directly from mouth to mouth, of masticated food such as fish or poi; infants and the aged were fed thus (Pukui and Elbert 1986, p. 344)), then the intimate nature of the relationship between an elder and the one providing them care becomes more specific. It also becomes clearer as to why it may be important for the relationship between the elder and caregiver to be close, as with a parent–child or grandparent–grandchild relationship. This importance is stressed in the following ‘Ōlelo No’eau:

**Hana ‘ino i kā ke kino ‘elemakule a ho’omakua aku i kā ha’i.** Mistreat your own oldsters and the day may come when you’ll be caring for someone else’s.

Said to a rude or ungrateful child (or grandchild). You should think of your own elder first, while he is alive, lest after his death you must take care of someone who had no part in rearing you.

(Pukui [1983] 1997, p. 55, #454; Handy and Pukui [1958] 1998, p. 180)

In the worst-case scenario, if an elder has not raised children, they may find themselves without care in their old age. The direct translation of the following ‘Ōlelo No’eau articulates this situation, though Pukui’s interpretation that follows reframes it in the positive by which one who raises children finds themselves with care in their later life.

**‘Elemakule kama ‘ole moe i ke ala.** An oldster who has never reared children sleeps by the wayside.

Caring for and rearing children results in being cared for in old age.

(Pukui [1983] 1997, p. 41, #337)

#### 3.4.4. The Importance of Carefully Translating English Words into ‘Ōlelo Hawai’i

Living languages, like the Hawaiian language, are in a state of constant change. In 1987, the first Kōmike Hua ‘Ōlelo (Hawaiian Lexicon Committee) was formed to create words for concepts and material culture unknown to our Hawaiian ancestors (Kōmike Hua’ōlelo 2003). In 2003, the latest version of Māmaka Kaiao, a dictionary of such words identified from the 1980s to 2000, was published. It included words that do not appear in earlier dictionaries (Pukui and Elbert 1986; Andrews 1865) which provide invaluable information, but only cover Hawaiian vocabulary from the earliest days until the 1980s (Kōmike Hua’ōlelo 2003, p. xvii). Despite the admirable efforts of the Kōmike Hua ‘Ōlelo, the undertaking of creating words for new concepts and material culture unknown to our Hawaiian ancestors continues to be an ongoing task, as such new words are seemingly innumerable.

In translating *Pōmai and Her Papa* into ‘Ōlelo Hawai’i, we found it necessary to create additional new words pertaining to Alzheimer’s Disease and dementia and to specific professions who aid in caring for those living with these conditions. However, the creation of new terms in ‘Ōlelo Hawai’i was not a task that we took lightly. Much thought went into creating these terms as to avoid any confusion with existing ‘Ōlelo Hawai’i words and concepts.

We followed similar protocols to the Kōmike Hua ‘Ōlelo in creating the new words for our translation. Primarily this involved either combining existing Hawaiian words to create a new word, or using loan words that Hawaiianize the orthography of a word from a non-Polynesian language (Kōmike Hua’ōlelo 2003, pp. xviii–xix). The use of loan words was intentional in creating the terms Ma’i ‘Alekahaima for Alzheimer’s Disease, and kemenekia for dementia to explicitly identify these conditions and to prevent any confusion that could have arisen had we attempted to make a new term by combining existing Hawaiian words. For example, we initially pondered using the term “Ma’i Poina”, which could be translated as a “memory loss disease or condition”, (ma’i, being defined in part as a sickness, disease, or ailment, and poina defined in part as to forget; forgotten) (Pukui and Elbert 1986, pp. 221, 337). But this would do little to distinguish it from amnesia or brain trauma. Additionally, memory loss is only one of many other signs of dementia and Alzheimer’s, and focusing specifically on this singular aspect does not totally encapsulate the impacts of these conditions. Therefore, it was decided that loan words would be the most precise way to identify Alzheimer’s Disease and dementia and to differentiate it from other conditions.

For the rest of the terms created, the primary challenge was in using existing Hawaiian words to create new terms that would not be confused with other existing Hawaiian language concepts. For example, the term we used for Social Worker is Lawehana Kāko’o Kaiāulu (worker that supports the community). Using a term like “kanaka kāko’o kaiāulu” could cause confusion because the word “kanaka” (person) does not differentiate a trained professional Social Worker from any good person, say a volunteer, who contributes any manner of good deeds to society.

Each new term we created for our translation followed the thought process above by ensuring that: 1. There was no term currently in dictionaries available for the concept we were trying to describe; 2. The grammatical structure and definitions of our newly created terms were consistent with the definitions of words selected as the components of these new terms; 3. Our newly created terms were easily differentiated from other existing words, terms, and concepts in ‘Ōlelo Hawai’i.; and 4. Our newly created terms may not be confused with other English-language words, terms, and concepts for which Hawaiian language words may or may not already exist. In total, nine new terms were created for this translation, two of which were loan words, and seven that were translations consisting of combinations of existing hua ‘Ōlelo Hawai’i (see Table 2).

## 4. Shifting Power Structures Within Hā Kūpuna at UH Mānoa to Support ‘Ōlelo Hawai’i Revitalization Projects

To sponsor projects like these that support the revitalization of ‘Ōlelo Hawai’i and Hawaiian worldviews, university departments and programs must make significant changes that challenge and decolonize existing power structures (Appleton 2019). Typically, a department chair or principal investigator would oversee important aspects of a project’s development, including identifying its objectives, conceptualizing research frameworks, identifying specific tasks and timelines, and creating and editing portions of the work itself. However, Hā Kūpuna’s current principal investigator, who is not Native Hawaiian and who does not speak ‘Ōlelo Hawai’i, was not able to participate substantively in many aspects of the development, creation, and execution processes for the Hawaiian language projects described in this paper. Though she was able to participate in setting timelines and editing English-language portions of our projects, she needed to trust her ‘Ōlelo Hawai’i-speaking staff to lead most aspects of these projects.

To aid in providing content-specific guidance to this staff, an ‘Ōlelo Hawai’i mentor from another college, who also serves as a mentor for the Ke’ena Noi’i a Unuhi ‘Ōlelo Hawai’i, was contracted. This mentor provided invaluable guidance in the form of language expertise, Hawaiian language research methodologies, and editing ‘Ōlelo Hawai’i documents produced, while the Hā Kūpuna staff remained primarily responsible for identifying the objectives of our projects, identifying and selecting research frameworks, identifying specific tasks and timelines, and executing and editing the final documents.

Hā Kūpuna decided to embark on Hawaiian language projects as a way to demonstrate how non-language-focused academic units can help in our university’s mission to become a Hawaiian Place of Learning. Helpful strategies in this process include hiring and steering Indigenous students into PhD programs, helping Indigenous PhD graduates secure faculty positions, and mentoring Indigenous junior faculty toward leadership roles. Also critical are ongoing and frank discussions of White privilege, racial identify and management, power dynamics, and social justice (Rodríguez et al. 2021; Martin-McDonald and McCarthy 2008). Hā Kūpuna also is committed to promoting respectful and collaborative methods in its qualitative and quantitative research projects (Haitsuka et al. 2023).

## 5. Conclusions

‘Ōlelo Hawai’i has experienced high levels of fluency and literacy in the Hawaiian Kingdom era followed by a traumatic loss in speakers and the current resurgence and revitalization of the language and its speakers and writers. Hā Kūpuna has benefited from decades of contributions from contemporary scholars and organizations who have made over a century’s worth of knowledge more easily accessible. Building upon their hard work, we have carved a place for ‘Ōlelo Hawai’i projects to be conducted within one more department in the University of Hawai’i System. Making space for this to occur has required a shift in power, with our principal investigator placing great trust and large responsibility in her ‘Ōlelo Hawai’i-speaking staff to create, execute, and complete the projects described in this paper. These projects have yielded insights into ancestral Hawaiian perspectives on elders and eldercare. These insights include gaining clarity on the evolution of the usage of the word “kupuna”, which is the hua ‘Ōlelo Hawai’i (Hawaiian word) used for elders today, identifying nuanced perspectives on elders that run counter to the predominantly held reverent notions of elders, understanding the importance of family as it relates to caregiving, and understanding the importance of carefully translating English words into ‘Ōlelo Hawai’i.

Though our work in expanding our understanding of ancestral Hawaiian perspectives on elders has just begun, it is an important and incremental contribution to future elder and eldercare research into Hawaiian language resources by organizations and other scholars. The creation of these collections demonstrates the continued practice of looking to our ancestors for guidance; such that future generations will know that in 2023, we still held such cultural values. This work is already gaining great interest from the public, with many organizations, individuals, and families requesting copies. We generated early interest for the glossary and ‘Ōlelo No’eau collections with a free online webinar hosted by Papa Ola Lōkahi (a non-profit consortium of organizations and public institutions that work on issues related to Native Hawaiian health and wellness) in December 2021. In the intervening years, our mentor Kumu Kapali Lyon directed us to consult with Dr. Kekeha Solis who published a 2010 dissertation on the topic of ‘Ōlelo No’eau. In the abstract of that dissertation Dr. Solis articulates, “Our kūpuna were able to utilize and manipulate proverbs to deal with a new era. And this is an important idea to build on in our language revitalization movement” (p. viii) (Solis 2010). It is our hope that the two collections will be used as reference materials for Hawaiian language speakers of all levels so that the philosophy and worldview expressed in ‘Ōlelo Hawai’i vocabulary and sayings begin to reappear in the imagination and speech of Kānaka Maoli and kama’āina in Hawai’i today. The completed booklets were ready for dissemination in 2023, with dozens of people requesting via google form to be notified when they were ready. To date we have distributed 149 hard copies each of our glossary and ‘Ōlelo No’eau collections, with an unknown number of digital copies of these materials downloaded from our website. Many of the requests for copies have been accompanied by family stories of language loss and excitement to reconnect themselves (and often their grandchildren) with our language. By the end of 2025, more than 1500 copies of the English version of *Pōmai and Her Papa* and 500 copies of the Hawaiian language version *Pōmai Lāua ‘o Papa* will have been distributed to every public and school library in the state. Normalizing the Hawaiian language, even on library shelves, is an important way for Native Hawaiians to reclaim public spaces in their homeland. We remain thankful to all of our Kumu ‘Ōlelo Hawai’i (Hawaiian language teachers), our Kumu ‘Ike Hawai’i (Hawaiian knowledge teachers), all kūpuna, luāhine, ‘elemākule past, present, and future, our mentors and staff, and to all others who continue to learn our language and learn about our language and culture and continue to increase our understanding of ancestral knowledge as we navigate the nuances of our living culture. May we all continue to reconnect with this knowledge as it guides us forward.

**Mai kāpae i ke a’o a ka makua, aia he ola ma laila.** *Do not ignore the teachings of a parent, for there is life in them*.

(Pukui [1983] 1997, p. 224, #2065; Handy and Pukui [1958] 1998, p. 194)

## Figures and Tables

**Table 1.** Enrollment in Hawaiian Schools and English Schools from 1848 to 1902 in approximately 20-year intervals. Data taken directly from Reinecke (1969, pp. 70–72).

Year	Total Schools	Total Students	Schools Conducted in Hawaiian	Students Enrolled in Hawaiian Schools	% of Total Enrollment	Schools in (or Offering) English	Students in English Schools	% of Total Enrollment
1848	631	19,844	624	19,644	99.0	7	200	1.0
1868	266	8404	221	6323	75.2	45	2081	24.8
1888	179	8770	63	1370	15.7	116	7400	84.3
1897	192	14,522	1	26	0.2	191	14,496	99.8
1902	203	18,382	0	0	0.0	203	18,382	100.0

Gray color on table is used to highlight statistics specific to Hawaiian schools and to make the left and right, and middle portions of the table easy to distinguish from each other and to read.

**Table 2.** New ‘Ōlelo Hawai’i Terms Created for the ‘Ōlelo Hawai’i Translation of Pōmai Lāua ‘o Papa.

English Term	‘Ōlelo Hawai’i Term Used	‘Ōlelo Hawai’i Term Type
**Alzheimer’s Disease**	Ma’i ‘Alekahaima	Loan Word
**Counselor**	Mea A’oa’o	Translation
**Dementia**	Kemenekia	Loan Word
**Geriatrician**	Kauka kilo mākule	Translation
**Health Plan**	Palapala Ho’olālā Ola Pono	Translation
**Neurologist**	Kauka kilo lolo	Translation
**Peer Educator**	Hoa Ho’ona’auao	Translation
**Progressive Disease**	Ma’i Māhua Mau	Translation
**Social Worker**	Lawehana kāko’o kaiāulu	Translation

## Data Availability

Research products are freely downloadable from https://manoa.hawaii.edu/hakupuna/.
